# Estimated Maternal Pesticide Exposure from Drinking Water and Heart Defects in Offspring

**DOI:** 10.3390/ijerph14080889

**Published:** 2017-08-08

**Authors:** Jihye Kim, Michael D. Swartz, Peter H. Langlois, Paul A. Romitti, Peter Weyer, Laura E. Mitchell, Thomas J. Luben, Anushuya Ramakrishnan, Sadia Malik, Philip J. Lupo, Marcia L. Feldkamp, Robert E. Meyer, Jennifer J. Winston, Jennita Reefhuis, Sarah J. Blossom, Erin Bell, A. J. Agopian, the National Birth Defects Prevention Study

**Affiliations:** 1 Department of Epidemiology, Human Genetics and Environmental Sciences, University of Texas School of Public Health, Houston, TX 77030, USA; Jihye.Kim416@gmail.com (J.K.); Laura.E.Mitchell@uth.tmc.edu (L.E.M.); 2 Department of Biostatistics, University of Texas School of Public Health, Houston, TX 77030, USA; Michael.D.Swartz@uth.tmc.edu; 3 Birth Defects Epidemiology and Surveillance Branch, Texas Department of State Health Services, Austin, TX 78714, USA; peter.langlois@dshs.texas.gov; 4 Department of Epidemiology, College of Public Health, University of Iowa, Iowa City, IA 52242, USA; paul-romitti@uiowa.edu; 5 Center for Health Effects of Environmental Contamination, University of Iowa, Iowa City, IA 52242, USA; peter-weyer@uiowa.edu; 6 National Center for Environmental Assessment, United States Environmental Protection Agency, Research Triangle Park, NC 27711, USA; Luben.Tom@epa.gov; 7 Community Epidemiology and Health Planning Unit, Cook County Department of Public Health, Oak Forest Health Center, Oak Forest, IL 60452, USA; anushuyar@gmail.com; 8 Department of Pediatrics, University of Texas, Southwestern, Dallas, TX 75390, USA; Sadia.Malik@UTSouthwestern.edu; 9 Department of Pediatrics, Baylor College of Medicine, Houston, TX 77030, USA; philip.lupo@bcm.edu; 10 Division of Medical Genetics, Department of Pediatrics, University of Utah School of Medicine, Salt Lake City, UT 84108, USA; Marcia.Feldkamp@hsc.utah.edu; 11 Division of Public Health, Birth Defects Monitoring Program, State Center for Health Statistics, Raleigh, NC 27699, USA; robert.meyer@dhhs.nc.gov; 12 Department of Obstetrics and Gynecology, University of North Carolina, Chapel Hill, NC 27599, USA; jen_winston@med.unc.edu; 13 National Center on Birth Defects and Developmental Disabilities, CDC, Atlanta, GA 30329, USA; nzr5@cdc.gov; 14 Department of Pediatrics, University of Arkansas for Medical Sciences, Arkansas Children’s Hospital Research Institute, Little Rock, AR 72202, USA; blossomsarah@uams.edu; 15 Department of Environmental Health Sciences, University at Albany School of Public Health, Albany, NY 12144, USA; ebell@albany.edu

**Keywords:** pesticide, congenital heart defect, public drinking water, Texas, birth defects

## Abstract

Our objective was to examine the relationship between estimated maternal exposure to pesticides in public drinking water and the risk of congenital heart defects (CHD). We used mixed-effects logistic regression to analyze data from 18,291 nonsyndromic cases with heart defects from the Texas Birth Defects Registry and 4414 randomly-selected controls delivered in Texas from 1999 through 2005. Water district-level pesticide exposure was estimated by linking each maternal residential address to the corresponding public water supply district’s measured atrazine levels. We repeated analyses among independent subjects from the National Birth Defects Prevention Study (NBDPS) (1620 nonsyndromic cases with heart defects and 1335 controls delivered from 1999 through 2005). No positive associations were observed between high versus low atrazine level and eight CHD subtypes or all included heart defects combined. These findings should be interpreted with caution, in light of potential misclassification and relatively large proportions of subjects with missing atrazine data. Thus, more consistent and complete monitoring and reporting of drinking water contaminants will aid in better understanding the relationships between pesticide water contaminants and birth defects.

## 1. Introduction

Congenital heart defects (CHDs) are among the most common congenital malformations, occurring in nearly 1% of live births [1]. CHDs account for approximately one-third of birth defects and are a leading cause of infant mortality due to birth defects [2,3]. The etiology of CHDs is poorly understood in the majority of nonsyndromic CHD cases (i.e., those that do not occur due to a recognized genetic syndrome). The prevalence of these defects varies by subtype of CHD [4], geography [5], and season of conception [6,7], which may suggest the involvement of environmental risk factors, such as pesticides, that also vary by geography and season.

In animal studies, some pesticides (e.g., organo-phosphorous pesticides, glyphosate) have been shown to have reproductive toxicity and teratogenic effects [8,9]. Observational studies in humans have reported associations between CHDs in offspring and parental occupational pesticide exposure [10], as well as residential proximity to agricultural areas where pesticides are applied [11,12,13]. Similar associations have been observed with other birth defects [14,15,16,17].

Water contamination may be an important source of human pesticide exposure, and higher versus lower maternal residential tap water consumption has been associated with CHDs in offspring [18]. The pesticide atrazine is of particular interest because it is one of the most widely applied agricultural pesticides in the U.S. [19], is present at substantial levels in ground water supplies (e.g., ≥0.003 mg/L, the U.S. Environmental Protection Agency (EPA) drinking water standard) [20,21], and is suspected to have adverse reproductive effects [21]. However, to our knowledge, no published study has evaluated atrazine or other pesticides in drinking water and CHD risk. In fact, we are aware of only three studies that have assessed associations between pesticides in water and any birth defect [22,23,24]. To address this gap, we examined the association between estimated maternal atrazine exposure via residential water district data from the EPA and risk for CHDs in offspring, using two independent data sources, the Texas Birth Defects Registry, and the National Birth Defects Prevention Study.

## 2. Materials and Methods

### 2.1. Texas Birth Defects Registry

The Texas Birth Defects Registry is an active, ongoing, population-based registry of birth defects that is maintained by the Texas Department of State Health Services Birth Defects Epidemiology and Surveillance Branch. Information on the Registry’s data collection methods has been described in a previous paper [25]. Briefly, trained Registry staff review records at birthing centers, midwife facilities, and hospitals statewide to identify cases with birth defects (live births, still births, or induced pregnancy terminations). Cases reported in the Registry must have a diagnosis of a structural birth defect or chromosome abnormality within one year of delivery to a mother residing in Texas at the time of birth. Registry staff abstract medical charts for each case, review case diagnoses, and assign a modified British Pediatric Association (BPA) code for each diagnosis [26]. Registry data are also linked to vital records (birth and fetal death certificates) from the Vital Statistics Unit of the Texas Department of State Health Services. These data were obtained and the analyses were conducted with approval from the Institutional Review Boards of the University of Texas Health Science Center at Houston and the Texas Department of State Health Services.

In this study, cases were defined as subjects with any structural CHD (BPA codes: 745.000–745.490, 745.510–745.900, 746.000–746.995, 747.100–747.320, 747.330–747.490) delivered from 1 January 1999 through 31 December 2008. We excluded births that only had a patent ductus arteriosus, patent foramen ovale, anomaly of umbilical artery, peripheral vascular system defect, pulmonary artery branch stenosis, or circulatory system anomaly. We further limited our analyses to those cases with documented diagnoses after delivery and no chromosome abnormality/syndrome. A total of 10,000 controls were randomly selected among all live born infants without known major malformations (i.e., not present in the Registry) delivered in Texas during the same study period. Subjects without a geocoded maternal address at delivery were excluded. Data on demographic and reproductive characteristics (i.e., potential confounders) were obtained from vital records. For the main analyses, cases and controls were restricted to subjects who were delivered from 1 January 1999 through 31 December 2005 (i.e., years with atrazine measurements, described below).

### 2.2. Water District Assignment

Our overall exposure assessment was based on linking each residential maternal address to the corresponding water supply district and estimating atrazine exposure based on measured levels for the district, as described below. The maternal address at delivery, as listed on vital records, was used as a proxy for address at conception. Only one address was reported for each mother. We obtained a map of geocoded boundaries for all public water systems in Texas from the Texas Water Development Board [27]. Each maternal address was converted to geocoded coordinates and these coordinates were overlaid with the water system map to identify the corresponding water district for each address.

### 2.3. Exposure Assessment

We obtained data on atrazine measurements from each water district’s routine monitoring during 1998–2005 from the Environmental Protection Agency (EPA) (https://www.epa.gov/dwsixyearreview/six-year-review-2-contaminant-occurrence-data-1998-2005) [28]. The EPA requires quarterly monitoring except under certain conditions when it allows reduced monitoring. If a water system meets certain criteria, EPA grants waivers from the regular monitoring (40 CFR Part 141.24) [29]. If the measurements are consistently below the detectable assay limit, EPA guidelines allow for less frequent testing [30]. Measurements at or below the limit of detection for the assay were coded as 0 µg/L for our main analysis, based on an approach proposed by Rinsky et al. [30]. Sensitivity analyses were also conducted, coding such measurements as the minimum detectable value for the assay instead. In our study, each subject was linked to atrazine measurements by matching residential addresses and public water districts. Because many districts did not have atrazine measurements at frequent and regular intervals (and in some instances measurements were not available during the period of the pregnancy, see Results), we used the mean of atrazine levels during the entire monitoring period (1999–2005) for each subject’s district. Based on the distributions in controls, mean atrazine values were categorized as “low” (<the 85th percentile) or “high” (≥the 85th percentile). For secondary analyses, the median atrazine levels were also categorized, using the same percentiles.

### 2.4. Statistical Analysis

Demographic characteristics of cases and controls in Texas who had atrazine information were compared using the 2-sided χ^2^ test. Because a large proportion of both cases and controls were missing atrazine levels (see Results), we also compared characteristics of those with and without atrazine data among all potential controls and compared the association between missing atrazine data and any CHD, using a 2-sided χ^2^ test. Odds ratios (ORs) and 95% confidence intervals (CIs) were also estimated for these comparisons.

We evaluated the association between high mean atrazine level and each of the eight CHD subtypes (e.g., conotruncal heart defects), as well as the combined group of any CHD (i.e., to assess the impact of missing atrazine measurements within population subgroups, see below and Results). To account for the correlation (i.e., clustering) between atrazine exposure and geographic location resulting from the use of a water district-level exposure assessment, mixed-effects (random-effects) models for logistic regression were used to investigate the association between maternal atrazine exposure and the risk of CHDs in offspring. Thus, we modeled subjects’ public water district as a random effect. We estimated crude and adjusted ORs and 95% CIs, using the lowest atrazine exposure category as the reference group. All models were adjusted for literature-based, a priori potential confounders that could be associated with atrazine and congenital heart defects: delivery year, maternal age at delivery (<20, 20–24, 25–29, 30–34, 35–39, and ≥40 years), race/ethnicity (non-Hispanic White, non-Hispanic Black, Hispanic, and other), education (less than high school, high school, and greater than high school), birth place (U.S. or outside of the U.S.), smoking (yes or no), and previous live birth (yes or no). In a secondary model, we adjusted for the population size served by the subject’s water district (<5000, 5000–200,000, and >200,000) and urban/rural status (metropolitan, non-metropolitan but urban, and less urbanized) because of large differences in the proportion of subjects missing atrazine data between categories of these variables. For all of our analyses, we did not conduct comparisons for CHD subgroups in which fewer than 50 water districts were represented among cases, due to concerns regarding model validity and convergence in mixed models with group-level sparseness [31,32].

To limit the potential for selection bias related to the exclusion of subjects without atrazine exposure data, we repeated our analysis of any CHD among subjects who lived in districts that served >200,000 people, because these larger districts had few subjects missing atrazine exposure. (Analyses of specific CHDs were not performed under this secondary model due to small numbers in some groups.) This analysis was repeated using the median atrazine level instead of the mean. To assess the extent of selection bias related to missing atrazine, we repeated the main analysis of any CHD with a 3-level exposure variable (i.e., low/high/missing). Additionally, we performed two sensitivity analyses for the analysis of any CHD: (1) excluding subjects from region 11 (south Texas), where over-ascertainment of CHDs is suspected [5,33], and (2) expanding to include subjects delivered from 1999–2008 (i.e., to improve power and analyze more recent years). All analyses were performed using SAS version 9.3 (SAS Institute, Cary, NC, USA).

### 2.5. National Birth Defects Prevention Study

We used the National Birth Defects Prevention Study (NBDPS) as an independent sample to further evaluate the relationship between atrazine and CHDs. These analyses were approved by the institutional review boards (IRBs) of each NBDPS site, the Centers for Disease Control and Prevention, and the University of Texas Health Science Center at Houston.

The NBDPS is a large population-based case-control study of major birth defects in ten U.S. states. The details of subject selection and data collection methods have been reported previously [34]. Briefly, a detailed computer-assisted telephone interview, which included questions about maternal exposures before and during pregnancy, was conducted among mothers of infants with and without birth defects. For the present study, potential cases included babies with any CHD ascertained by the NBDPS [35], among live births, stillbirths, and elective terminations. All cases had confirmation by echocardiography, cardiac catheterization, surgery, or autopsy [36]. Cases with chromosome abnormalities or syndromes were not included in the NBDPS. Controls were live-born infants without any major birth defect, randomly selected from vital records or hospital discharge information, in the same areas and in the same study periods from which cases were selected.

NBDPS cases and controls delivered from 1997–2005 were available for analysis. In our main analyses, only subjects delivered from 1 January 1999 through 31 December 2005 were included, to match the study period used in the Texas analyses. Our NBDPS analyses were restricted to subjects from Arkansas (statewide), Iowa (statewide), North Carolina (northern Piedmont region counties, deliveries starting in 2003), and Utah (statewide, deliveries starting in 2003) of the NBDPS. Subjects from several other sites were excluded due to a high proportion of subjects residing in water districts, for which atrazine data were not available from the EPA. Subjects in the NBDPS from Texas were excluded to avoid overlap with subjects in the Texas analyses.

Atrazine exposure was assessed based on the linkage of maternal address at conception to the water supply district and the reported atrazine levels for each district from the EPA. If multiple addresses were reported during the month before conception through the third month of pregnancy, we used the first (i.e., earliest) address. The public water supply districts for mothers were identified by overlaying addresses with digitized service area maps for public water supplies obtained from local state agencies or municipal water utilities for each state [37]. If use of the digitized maps failed to identify a subject’s district, the district was assigned based on census place boundaries or, in rare instances, using interactive searching methods [37].

Using the same methods as in our Texas analyses, we repeated our main analyses using NBDPS data. Because the NBDPS had some CHD subtypes with small numbers and had relatively lower mean atrazine levels compared to Texas (90th percentile in controls: 0.41 µg/L in Texas versus 0.08 µg/L in NBDPS), atrazine values were categorized using different cut-offs for NBDPS to make the distribution more comparable to Texas categories: “low” (<the 95th percentile) and “high” (≥the 95th percentile). We adjusted for the same potential confounders as in the Texas analyses, except for rural/urban status, which was not available in the NBDPS. We also included an additional secondary regression model, adjusting for two variables that were not available for Texas subjects: household income (<$10,000, $10,000–50,000, and >$50,000) and supplemental folic acid intake (yes or no). For comparison to the secondary analyses among Texas subjects, we also repeated any CHD analyses among subjects in water districts serving a population size >25,000. (Analyses among districts serving >200,000 people or for specific CHD subtypes among districts serving >25,000 people could not be performed due to small numbers.) We also repeated analyses for any CHD among subjects in water districts serving >25,000 people using the median atrazine level (≤50th percentile versus >50th percentile).

## 3. Results

### 3.1. Texas Birth Defects Registry

There were 43,512 infants with non-syndromic CHDs in Texas during 1999–2008. We randomly selected 10,000 controls among live-born infants statewide during this period. Out of 53,512 total subjects, 5889 (11.0% of cases and 11.1% of controls) did not fall within a public water system boundary (i.e., those with private wells) and were excluded. We also excluded 11,995 subjects (22.1% cases and 24.0% controls) who resided in water districts without any recorded atrazine levels during the monitoring period (1999–2005). Characteristics of these subjects were assessed (see below). Our main analyses were restricted to deliveries during 1999–2005 (i.e., years with atrazine measurements), resulting in 22,705 included subjects (18,291 cases and 4414 controls). (An additional 12,923 subjects delivered during 2006–2008 were included in a sensitivity analyses, described below.)

Frequencies and distributions for demographic and reproductive characteristics of cases and controls were tabulated (Table 1). There were significant differences between cases and controls in the distribution of maternal age, race/ethnicity, birthplace, pregestational or gestational diabetes, and plurality, as well as infant sex and delivery year. Further, there were differences in the distribution of residential factors between cases and controls, including the population size the water district served and the region of Texas where the mother resided.

In Texas, 26.1% of all potential controls resided in water districts without atrazine data. There was a significant association between missing atrazine data and any CHD (*p*-value < 0.001, data not shown). To better characterize these subjects, we compared the distributions of demographic and reproductive characteristics between potential control subjects (all live born infants without birth defects in Texas between 1999 and 2005) with and without atrazine data (Table 2). There were significant differences (*p*-value < 0.001) in the distributions of 11 of these 13 factors. The differences with the largest magnitudes of effects were maternal race/ethnicity and residential factors, including population size of the water district served, region in Texas, and urban/rural status. Of note, atrazine measurements were present for 94.6% of potential control subjects residing in districts serving >200,000 people.

In our main association analyses, we assessed eight specific CHD subtypes as well as any CHD (Table 3). In these analyses, there was a negative association between high versus low mean atrazine level and ventricular septal defects (AOR 0.8, 95% CI: 0.6, 1.0), after adjusting for delivery year, maternal age, race/ethnicity, education, birthplace, smoking, and history of previous live birth, and a similar result was observed after adjusting for water district population size and rural/urban status. The atrazine level was not significantly associated with any of the other seven CHD subtypes assessed or with any CHD in either adjusted model (range of adjusted odds ratios (AOR): 0.8–1.5). We repeated these analyses during an extended time period, for deliveries during 1999 through 2008, and these results were similar (data not shown). We repeated the main analysis for any CHD after excluding region 11 of Texas (where CHD cases may have been potentially over-ascertained) [33] and results were similar (data not shown).

To address the possibility of selection bias related to subjects in districts missing atrazine measurements, we also repeated the main analyses for any CHDs, restricting to subjects in water districts serving >200,000 people, as few subjects in these districts were missing atrazine exposure estimates. In these analyses (Table 4), high versus low median and mean atrazine levels were not significantly associated with any CHD. We also repeated the analyses shown in Table 3 and Table 4 after using the value for the minimal detectable limit (MDL) instead of 0 when a measurement was at or less than the MDL (97.3% of subjects did not change exposure category), and results from these analyses were similar to the main results (data not shown). We also repeated the main analyses for any CHDs using a three-level exposure variable (i.e., low, high, and missing), and the results for high versus low atrazine were similar to the main results (data not shown).

### 3.2. National Birth Defects Prevention Study

For comparison, we conducted similar analyses in the NBDPS for deliveries during 1999 through 2005. A total of 3591 subjects were available in the NBDPS and 636 subjects (8.7% of cases, 19.4% of controls) were excluded due to missing atrazine values, resulting in 2955 included subjects (1620 cases and 1335 controls). Demographic and reproductive characteristics were compared (Table S1), and differences were observed for state, delivery year, plurality, maternal age, maternal pregestational or gestational diabetes, and water district population size between cases and controls. In the NBDPS, 19.4% of control mothers were missing atrazine information, and differences in the distributions of state, delivery year, maternal age, household income, maternal pregestational or gestational diabetes, and water district population size were present between control subjects in districts with versus without atrazine measurements (Table S2). However, the distribution of missing atrazine data was not different between cases with any CHD and controls (*p*-value = 0.126)

We repeated our main association analyses in the NBDPS (Table 5). In these analyses, there was a negative association between high versus low mean atrazine level and left ventricular outflow tract defects (AOR 0.4, 95% CI: 0.2, 0.9) after adjusting for delivery year, maternal age, race/ethnicity, education, birthplace, smoking, and history of previous live birth. The magnitude of this result was similar after adjusting for water district population size, and after adjusting for potential confounding factors not present in the Texas data (i.e., household income and folic acid intake). The atrazine level was not significantly associated with any of the other six CHD subtypes assessed or with any CHD in either adjusted model (range of adjusted odds ratios (AOR): 0.5–1.2).

When limiting any CHD analyses to water districts serving >25,000 people to minimize potential selection bias in the NBDPS (11.2% of cases and 12.5% of control subjects missing atrazine), the results were similar to those from the main analysis in Table 5 (data not shown). When using the median atrazine level in these analyses, the results were also similar to those from Texas (data not shown).

## 4. Discussion

We examined the association between maternal exposure to atrazine in public drinking water and CHDs in offspring using two large data sources. In our main analyses for Texas (1999–2005), estimated high atrazine exposure in public drinking water was not positively associated with any of the eight CHD subtypes assessed or any CHD (i.e., the group of any CHDs). In fact, ventricular septal defects were negatively associated with atrazine in Texas. In follow-up analyses in the NBDPS (1999–2005), no positive associations were observed between atrazine and any of the seven CHD subtypes assessed or any CHD, and a negative association was observed with left ventricular outflow tract defects. The observed magnitude of effect was <1 for most comparisons within the NBDPS. Our findings were similar across several sensitivity analyses, and do not support a positive association between atrazine and CHDs. However, our findings should be interpreted in light of limitations of the data.

Similar to other studies that have used water-district level data for exposure estimates [24,38], the accuracy of our exposure estimates is uncertain. Given the relative rarity of birth defects, prospective studies with direct individual-level measurements are not feasible and exposure estimates may be the only available data. In the data we analyzed, there were a large proportion of water districts missing atrazine data, especially in Texas. As mothers in Texas districts with atrazine data had different distributions of several characteristics compared to those without atrazine data, it is possible that our analyses were impacted by selection bias, and that our results may not generalize to all women. However, when we accounted for subjects with missing data using a 3-level exposure variable (low/high/missing), the estimate for low versus high exposure did not change, which may suggest that there were not major effects due to missing exposure data.

We tried to further limit this potential selection bias by conducting sensitivity analyses among subjects in districts with more complete atrazine measurements (i.e., districts serving large populations), though these analyses could not be conducted for specific CHD subtypes due to small case numbers. We further tried to limit potential selection bias by using a secondary data source with more complete exposure data (i.e., the NBDPS), though substantially fewer cases were present among this data source. These limitations with the exposure data highlight the lack of complete collection and curation of public data on at least some pesticide water contaminants. It was unclear from these analyses if the atrazine data were missing because of districts not complying with mandatory reporting requirements, not properly reporting the data to the EPA, or another issue. Further, as several published studies have used water district monitoring data for similar analyses (e.g., other contaminants or outcomes), our findings emphasize the need for caution in interpreting results from such studies, which do not always address the impact of missing exposure data.

We observed negative associations between high versus low atrazine and two CHD subtypes: ventricular septal defects (VSDs) and left ventricular outflow tract (LVOT) defects. As we are not aware of any hypothesized protective effect of atrazine on birth defects (and these results were not entirely consistent between Texas and the NBDPS), we suspect that these observations may have arisen due to the limitations of the data (e.g., selection bias and/or measurement error). Alternatively, we cannot rule out the possibility that these trends might be explained by a relationship between atrazine and a very severe phenotype that resulted in an early fetal death or miscarriage (i.e., potential cases that were not ascertained). In fact, early fetal death/miscarriage has been associated with atrazine in animal [39] and human [40,41] studies.

Some previous studies have reported on the reproductive effects of atrazine. In animal studies, atrazine exposure has been associated with developmental effects, though relatively few of these studies have focused on malformations of the heart. Both zebrafish and rats have shown functional disturbances of the heart following atrazine exposure [42,43,44]. Another study observed that 23% of chicken embryos treated with *N*-nitrosoatrazine (which is formed when atrazine reacts with nitrite) had malformations, including CHDs [45].

Few studies have examined risks of CHDs in humans and pesticide exposure from water. Based on ecologic data, it was observed that Iowa communities served by a water reservoir contaminated with atrazine had higher rates of CHDs compared to other communities [46], but individual-level data were not available. Although a U.S. ecological study reported a correlation between surface water atrazine and birth defects overall [24], to our knowledge no prior analytic study has evaluated atrazine in water and individual-level risk of CHDs specifically.

However, other previous observational studies have reported links between atrazine in drinking water and non-CHD birth defects or reproductive outcomes. For example, a study from the NBDPS reported a weak association between atrazine (estimated based on catchment level stream and groundwater contaminant models) in drinking water and hypospadias [47]. In addition, a U.S. study observed an elevated prevalence of abdominal wall defects during months when seasonal patterns of atrazine and nitrate levels in surface water peaked [22], and in a semi-ecological study, the risk of gastroschisis was inversely associated with distance to the site of elevated atrazine exposure in surface water [23]. Some studies found that atrazine in drinking water was significantly associated with small for gestational age or preterm birth [30,48,49], whereas others did not find associations [50].

Although suspected effects of atrazine may be related to the combinations of multiple pesticides, agricultural compounds, or other chemicals in drinking water [21], few studies have assessed the effects of any of these water contaminants and CHDs. Chlorination byproducts, arsenic, and manganese in water have been independently associated with CHDs [51,52,53], and inconsistent results have been reported for nitrate in water and CHD risk [37,51,54].

In addition to water, occupational exposure is another important potential pesticide exposure source. Multiple studies have assessed occupational exposure to pesticides and CHDs, but the relationship is unclear. Briefly, Rocheleau et al. assessed maternal occupational exposure to pesticides and CHDs in the NBDPS [10] and found that maternal occupations with exposure to herbicides were associated with hypoplastic left heart syndrome, pulmonary valve stenosis, and tetralogy of Fallot. An additional case-control study conducted in Maryland found that mothers of cases with transposition of great arteries in offspring were more likely to use pesticides (at home, work, or other site) before or during pregnancy than mothers of controls [13]. However, a case-control study in China observed that maternal occupational exposure to pesticides during the periconceptional period was not associated with simple isolated CHDs [55], and another case-control study from California in U.S. also reported no association, but it had very few exposed cases (*n* = 7) [56].

Several studies have also assessed residential proximity to sites with agricultural pesticide application exposure and CHDs, although results have also been inconsistent. A population-based, case-control study observed that mothers residing near an area with agricultural pesticide application were more likely to have specific CHD subtypes in offspring than mothers who did not [11]. Another case-control study in North Carolina found that mothers exposed to high levels of agricultural pesticides near their residence had elevated risk of CHDs compared to mothers exposed to low levels of those pesticides [12]. Conversely, a registry-based, case-control study from Washington State revealed that neither higher proportions of agricultural land in the mother’s residence county nor agriculture-related maternal occupation were associated with the risk of ventricular septal defects [57].

Strengths of the present study include the use of population-based data, the large numbers of study subjects, the data on many potential covariates, the restriction to nonsydromic cases, the case classification review by clinicians (all NBDPS cases and a subset of Texas cases), and the repetition of similar analyses across independent data sources. However, there are limitations that need to be considered in the interpretation of our findings. Due to relatively sparse data on exposure levels over time for many districts (e.g., 26.1% of Texas control women’s districts were missing atrazine levels), we could not restrict the exposure time period to the critical period for heart development during early pregnancy. Instead, we used representative values of atrazine levels for each water district (e.g., mean) over the entire six-year monitoring period to define high or low atrazine groups, which may not have reflected atrazine levels during pregnancy (e.g., annual/seasonal changes were not reflected). Such a lack of seasonal specificity in atrazine levels might have led to measurement errors in this study, which may have biased effects towards the null. Also, the maternal residential address at conception was available in the NBDPS data, but not in the Texas data. As such, we used the address at delivery as a proxy in the Texas data. Results from previous studies assessing residential mobility during pregnancy in Texas and elsewhere suggest that this was unlikely to have made a major impact [58,59]. However, since we cannot ignore a possibility of measurement error due to the use of proxy addresses, formal measurement error modeling (e.g., Berkson model) would be of interest in future work.

As individual-level exposure data were not available, maternal atrazine exposure was estimated using ecologic-level data (water districts), and, similarly to other studies that have used water district data, we cannot rule out that an effect related to the ecological fallacy influenced our findings. To address this, we used mixed-effects models to take into account the latent correlation of atrazine levels between subjects within the same district. However, we cannot rule out the possibility of exposure misclassification related to atrazine measurement differences within or between water systems (e.g., season or frequency), which might have resulted in bias. Our main analyses also did not account for variability in water use and consumption habits (e.g., not consuming tap water at home), although there are future planned analyses to address these habits in the NBDPS. Further, it is not clear if the relatively low ranges of atrazine levels measured in this study were sufficiently large to potentially produce a detectable effect.

Although we used two of the largest sources of birth defects data worldwide, small numbers among specific CHD subtypes did not allow for some of our sensitivity analyses to be conducted among these subgroups. Instead, we conducted these analyses among cases with any CHD, though these secondary analyses may have been limited by etiologic heterogeneity.

## 5. Conclusions

Our study examined the association between estimated maternal atrazine exposure in drinking water and the risk of CHD subtypes in offspring. No positive associations were observed between atrazine and CHDs, though our findings should be interpreted in light of the limitations of our study. More consistent monitoring, reporting, and curation of pesticide water contaminants in public water sources might aid in further characterizing potential associations. Considering the current limitations of water district data and with the absence of relevant biomarkers of exposure, our findings suggest that use of other data sources as proxies for water contaminant exposure (e.g., proximity to pesticide application sites) may be helpful. If environmental contaminant measurements from maternal/infant blood samples become available in future studies, this would also be beneficial.

## Figures and Tables

**Table 1:** Descriptive characteristics of cases and controls with available atrazine data, Texas, 1999–2005.

Characteristic ^a^	Cases		Controls		*p*-Value ^b^
	*N*	%	*N*	%	
Total	18,291	100.0	4,414	100.0	
Delivery year					<0.001
1999	2027	11.1	570	12.9	
2000	2187	12.0	662	15.0	
2001	2362	12.9	643	14.6	
2002	2534	13.9	617	14.0	
2003	2698	14.8	633	14.3	
2004	3052	16.7	640	14.5	
2005	3431	18.8	649	14.7	
Infant sex					0.002
Male	9476	51.8	2,174	49.3	
Female	8814	48.2	2,240	50.8	
Plurality of pregnancy					<0.001
Yes	1190	6.5	106	2.4	
No	17,100	93.5	4,308	97.6	
Previous live birth					0.253
Yes	10,836	61.5	2,575	60.6	
No	6772	38.5	1,675	39.4	
Maternal age					<0.001
<20	2516	13.8	674	15.3	
20–24	4947	27.1	1,336	30.3	
25–29	4643	25.4	1,132	25.7	
30–34	3645	19.9	841	19.1	
35–39	1982	10.8	350	7.9	
≥40	554	3.0	80	1.8	
Maternal race/ethnicity					<0.001
Non-Hispanic White	5465	29.9	1,349	30.6	
Non-Hispanic Black	2104	11.5	567	12.9	
Hispanic	10,290	56.3	2,348	53.3	
Other	411	2.3	140	3.2	
Mother’s education					0.523
Less than high school	6301	35.0	1,498	34.5	
High school	5646	31.3	1,342	30.9	
Greater than high school	6084	33.7	1,506	34.7	
Mother’s birthplace in US					<0.001
Yes	12,861	70.7	2,985	67.9	
No	5326	29.3	1,413	32.1	
Maternal cigarette smoking					0.201
Yes	1052	5.8	231	5.3	
No	17,146	94.2	4,142	94.7	
Maternal gestational or pregestational diabetes					<0.001
Yes	1190	6.5	136	3.1	
No	17,014	93.5	4,278	96.9	
Water district population size					<0.001
<5000	4695	25.7	1,165	26.4	
5000–200,000	4261	23.3	901	20.4	
>200,000	9334	51.0	2,348	53.2	
Texas Public Health region					<0.001
1	901	4.9	186	4.2	
2	426	2.3	98	2.2	
3	4342	23.7	1,033	23.4	
4	329	1.8	140	3.2	
5	290	1.6	102	2.3	
6	2845	15.6	995	22.5	
7	1423	7.8	465	10.5	
8	2321	12.7	553	12.5	
9	554	3.0	122	2.8	
10	585	3.2	229	5.2	
11	4275	23.4	491	11.1	
Rural/urban status					0.157
Metropolitan, urbanized	16,808	91.9	4,041	91.6	
Non-metropolitan, urban	659	3.6	147	3.3	
Less urbanized	824	4.5	226	5.1	

**^a^** Some characteristic counts do not sum to the totals due to missing data; **^b^** estimated using chi-square test.

**Table 2:** Descriptive characteristics of potential controls with and without atrazine data in Texas, 1999–2005.

Characteristic ^a^	Atrazine Data		Missing Atrazine Data		OR (95%CI)	*p*-Value ^b^
	*N*	%	*N*	%		
Total	1,585,927	100.0	561,149	100.0		
**Delivery year**						**<0.001**
1999	215,516	13.6	71,562	12.8	Ref.	
2000	222,837	14.1	74,337	13.3	1.01 (0.99–1.02)	
2001	222,426	14.0	77,646	13.8	**1.05 (1.04–1.06)**	
2002	228,332	14.4	80,270	14.3	**1.06 (1.05–1.07)**	
2003	227,179	14.3	82,001	14.6	**1.09 (1.07–1.10)**	
2004	233,153	14.7	86,068	15.3	**1.11 (1.10–1.13)**	
2005	236,484	14.9	89,265	15.9	**1.14 (1.12–1.15)**	
**Infant sex**						0.939
Male	805,690	50.8	285,111	50.8	Ref.	
Female	780,237	49.2	276,038	49.2	1.00 (0.99–1.01)	
**Plurality of pregnancy**						**<0.001**
Yes	42,004	2.7	17,657	3.2	**1.19 (1.17–1.22)**	
No	1,543,831	97.4	543,463	96.9	Ref.	
**Previous live birth**						**<0.001**
Yes	934,055	61.1	328,080	60.3	**0.97 (0.96–0.98)**	
No	595,729	38.9	215,725	39.7	Ref.	
**Maternal age**						**<0.001**
<20	241,094	15.2	62,557	11.2	**0.70 (0.69–0.71)**	
20–24	464,051	29.3	136,582	24.3	**0.79 (0.79–0.80)**	
25–29	418,122	26.4	155,022	27.6	Ref.	
30–34	303,334	19.1	134,909	24.0	**1.20 (1.19–1.21)**	
35–39	131,809	8.3	60,184	10.7	**1.23 (1.22–1.25)**	
≥40	27,403	1.7	11,837	2.1	**1.17 (1.14–1.19)**	
**Race/ethnicity**						**<0.001**
Non-Hispanic White	488,010	30.8	287,212	51.3	Ref.	
Non-Hispanic Black	195,843	12.4	60,380	10.8	**0.52 (0.52–0.53)**	
Hispanic	849,795	53.7	178,962	32.0	**0.36 (0.36–0.36)**	
Other	49,929	3.2	33,506	6.0	**1.14 (1.12–1.16)**	
**Education**						**<0.001**
Less than high school	548,329	35.1	130,606	23.7	**0.72 (0.71–0.72)**	
High school	473,853	30.3	158,530	28.7	Ref.	
Greater than high school	540,961	34.6	262,870	47.6	**1.45 (1.44–1.46)**	
**Mother’s birthplace in US**						**<0.001**
Yes	1,079,009	68.0	411,507	73.3	Ref.	
No	506,918	32.0	149,642	26.7	**0.77 (0.77–0.78)**	
**Cigarette smoking during pregnancy**						**<0.001**
Yes	84,209	5.4	36,924	6.6	**1.25 (1.24–1.27)**	
No	1,489,334	94.7	521,828	93.4	Ref.	
**Maternal gestational or pregestational diabetes**						0.939
Yes	46,557	2.9	16,462	2.9	1.00 (0.98–1.02)	
No	1,539,369	97.1	544,686	97.1	Ref.	
**Water district population size**						**<0.001**
<5000	417,393	26.3	362,324	64.6	Ref.	
5000–200,000	327,863	20.7	150,244	26.8	**0.53 (0.52–0.53)**	
>200,000	840,649	53.0	48,366	8.6	0.07 (0.07–0.07)	
**Texas Public Health region**						**<0.001**
1	66,241	4.2	2,313	0.4	**0.05 (0.05–0.05)**	
2	34,809	2.2	7,143	1.3	**0.30 (0.29–0.30)**	
3	371,655	23.4	258,949	46.2	Ref.	
4	45,544	2.9	18,231	3.3	**0.58 (0.56–0.59)**	
5	36,130	2.3	9,074	1.6	**0.36 (0.35–0.37)**	
6	358,067	22.6	171,153	30.5	**0.67 (0.68–0.69)**	
7	174,704	11.0	55,537	9.9	**0.46 (0.45–0.46)**	
8	196,342	12.4	14,649	2.6	**0.11 (0.11–0.11)**	
9	40,897	2.6	1,164	0.2	**0.04 (0.04–0.04)**	
10	82,199	5.2	6,603	1.2	**0.12 (0.11–0.12)**	
11	179,339	11.3	16,333	2.9	**0.13 (0.13–0.13)**	
**Urban/rural status**						**<0.001**
Metropolitan, urbanized	1,451,694	91.5	520,544	92.8	Ref.	
Non-metropolitan, urban	57,631	3.6	12,773	2.3	**0.62 (0.61–0.63)**	
Less urbanized	76,602	4.8	27,832	5.0	**1.01 (1.00–1.03)**	

OR, (crude) odds ratio for missing atrazine; CI, confidence interval; **^a^** some characteristic counts do not sum to the totals due to missing data; **^b^** estimated using chi-square test.

**Table 3:** Association between estimated high mean atrazine in drinking water and heart defects in offspring in Texas, 1999–2005.

Title	Controls	Cases	OR (95% CI)	AOR ^a^ (95% CI)	AOR ^b^ (95% CI)
	*N* (%)	*N* (%)			
**Any CHD**					
Low mean atrazine	3,755 (85.1)	16,009 (87.5)	Ref.	Ref.	Ref.
High mean atrazine ^c^	659 (14.9)	2282 (12.5)	0.83 (0.65–1.06)	0.84 (0.66–1.06)	0.83 (0.65–1.05)
**ASD**					
Low mean atrazine	3,755 (85.1)	4,081 (87.3)	Ref.	Ref.	Ref.
High mean atrazine ^c^	659 (14.9)	593 (12.7)	0.97 (0.69–1.37)	0.95 (0.67–1.36)	0.96 (0.68–1.35)
**VSD**					
Low mean atrazine	3,755 (85.1)	6,271 (87.5)	Ref.	Ref.	Ref.
High mean atrazine ^c^	659 (14.9)	894 (12.5)	**0.73 (0.57–0.94)**	**0.75 (0.58–0.95)**	**0.73 (0.57–0.94)**
**AVSD**					
Low mean atrazine	3,755 (85.1)	103 (88.0)	Ref.	Ref.	Ref.
High mean atrazine ^c^	659 (14.9)	14 (12.0)	0.77 (0.44–1.36)	0.84 (0.47–1.49)	0.80 (0.45–1.41)
**CTD**					
Low mean atrazine	3,755 (85.1)	782 (86.2)	Ref.	Ref.	Ref.
High mean atrazine ^c^	659 (14.9)	125 (13.8)	0.87 (0.66–1.15)	0.91 (0.69–1.20)	0.87 (0.66–1.15)
***Tetralogy of Fallot***					
Low mean atrazine	3,755 (85.1)	359 (85.3)	Ref.	Ref.	Ref.
High mean atrazine ^c^	659 (14.9)	62 (14.7)	0.98 (0.74–1.31)	1.02 (0.75–1.38)	1.00 (0.75–1.32)
**LVOT**					
Low mean atrazine	3,755 (85.1)	133 (81.6)	Ref.	Ref.	Ref.
High mean atrazine ^c^	659 (14.9)	30 (18.4)	1.29 (0.84–1.97)	1.31 (0.86–1.99)	1.29 (0.84–1.97)
**RVOT**					
Low mean atrazine	3,755 (85.1)	416 (79.9)	Ref.	Ref.	Ref.
High mean atrazine ^c^	659 (14.9)	105 (20.2)	1.30 (0.91–1.86)	1.32 (0.91–1.92)	1.31 (0.92–1.86)
***Pulmonary valve stenosis***					
Low mean atrazine	3,755 (85.1)	348 (78.0)	Ref.	Ref.	Ref.
High mean atrazine ^c^	659 (14.9)	98 (22.0)	1.46 (0.98–2.16)	1.47 (0.98–2.21)	1.47 (1.00–2.16)

CHD, congenital heart defect; OR, (crude) odds ratio (odds of cases between the exposed group to high mean atrazine and the unexposed group); AOR, adjusted odds ratio; ASD, atrial septal defect; VSD, ventricular septal defect, AVSD, atrioventricular septal defect; CTD, conotruncal heart defect; LVOT, left ventricular outflow tract; RVOT, right ventricular outflow tract; **^a^** adjusted for delivery year, maternal age, race/ethnicity, education, birthplace, smoking, and history of previous live birth; **^b^** adjusted for population size of water district and rural/urban status; ^c^ “high mean atrazine” defined as ≥85th percentile (0.16 µg/L) of mean values across all atrazine measurements in controls, 1999–2005; otherwise, “low mean atrazine”.

**Table 4:** Association between estimated atrazine exposure in drinking water and congenital heart defects in offspring in Texas, 1999–2005, among districts with population size >200,000.

Any CHD	Controls	Cases	OR (95% CI)	AOR ^a^ (95% CI)
	*N* (%)	*N* (%)		
**High median atrazine**				
No (<95th percentile ^b^)	1,904 (81.1)	7,766 (83.2)	Ref.	Ref.
Yes (≥95th percentile ^b^)	444 (18.9)	1,568 (16.8)	0.82 (0.34–1.99)	0.79 (0.32–1.91)
**High mean atrazine**				
No (<95th percentile ^b^)	1,904 (81.1)	7,766 (83.2)	Ref.	Ref.
Yes (≥95th percentile ^b^)	444 (18.9)	1,568 (16.8)	0.82 (0.34–1.99)	0.79 (0.32–1.91)

CHD, congenital heart defect; OR, (crude) odds ratio; AOR, adjusted odds ratio; **^a^** adjusted for delivery year, maternal age, race/ethnicity, education, birthplace, smoking, and history of previous live birth; **^b^** all percentiles were based on data from full controls, 1999–2005 (0.37 µg/L for 95th percentile median, 0.41 µg/L for 95th percentile mean).

**Table 5:** Association between estimated high mean atrazine in drinking water and heart defects delivered in NBDPS **^a^**, 1999–2005.

	Controls	Cases	OR (95% CI)	AOR ^b^ (95% CI)	AOR ^c^ (95% CI)	AOR ^d^ (95% CI)
	*N* (%)	*N* (%)				
**Any CHD**						
Low mean atrazine	1,249 (93.6)	1,542 (95.2)	Ref.	Ref.	Ref.	Ref.
High mean atrazine ^e^	86 (6.4)	78 (4.8)	0.64 (0.37–1.12)	0.60 (0.34–1.05)	0.65 (0.37–1.13)	0.64 (0.36–1.11)
**ASD**						
Low mean atrazine	1,249 (93.6)	364 (96.8)	Ref.	Ref.	Ref.	Ref.
High mean atrazine ^e^	86 (6.4)	12 (3.2)	0.50 (0.22–1.17)	0.47 (0.20–1.11)	0.52 (0.22–1.19)	0.53 (0.22–1.25)
**VSD**						
Low mean atrazine	1,249 (93.6)	247 (94.6)	Ref.	Ref.	Ref.	Ref.
High mean atrazine ^e^	86 (6.4)	14 (5.4)	0.56 (0.23–1.38)	0.55 (0.22–1.36)	0.58 (0.24–1.41)	0.46 (0.18–1.18)
**CTD**						
Low mean atrazine	1,249 (93.6)	226 (93.4)	Ref.	Ref.	Ref.	Ref.
High mean atrazine ^e^	86 (6.4)	16 (6.6)	0.89 (0.37–2.13)	0.79 (0.33–1.88)	0.99 (0.44–2.27)	0.95 (0.41–2.19)
***Tetralogy of Fallot***						
Low mean atrazine	1,249 (93.6)	102 (93.6)	Ref.	Ref.	Ref.	Ref.
High mean atrazine ^e^	86 (6.4)	7 (6.4)	0.89 (0.25–3.14)	0.85 (0.26–2.80)	1.15 (0.39–3.39)	0.90 (0.26–3.04)
**LVOT**						
Low mean atrazine	1,249 (93.6)	266 (97.1)	Ref.	Ref.	Ref.	Ref.
High mean atrazine ^e^	86 (6.4)	8 (2.9)	0.40 (0.16–1.04)	**0.36 (0.15–0.91**)	0.41 (0.16–1.07)	**0.38 (0.15–0.98)**
**RVOT**						
Low mean atrazine	1,249 (93.6)	250 (94.3)	Ref.	Ref.	Ref.	Ref.
High mean atrazine ^e^	86 (6.4)	15 (5.7)	0.69 (0.29–1.63)	0.67 (0.29–1.55)	0.74 (0.32–1.71)	0.70 (0.30–1.63)
***Pulmonary valve stenosis***						
Low mean atrazine	1,249 (93.6)	253 (94.8)	Ref.	Ref.	Ref.	Ref.
High mean atrazine ^e^	86 (6.4)	14 (5.2)	0.62 (0.25–1.51)	0.60 (0.25–1.45)	0.64 (0.26–1.53)	0.63 (0.26–1.54)

NBDPS, National Birth Defects Prevention Study; CHD, congenital heart defect; OR, (crude) odds ratio (odds of cases between the exposed group to high mean atrazine and the unexposed group); AOR, adjusted odds ratio; ASD, atrial septal defect; VSD, ventricular septal defect, AVSD, atrioventricular septal defect; CTD, conotruncal heart defect; LVOT, left ventricular outflow tract; RVOT, right ventricular outflow tract; **^a^** NBDPS included data from Arkansas, Iowa, North Carolina, and Utah; **^b^** adjusted for delivery year, maternal age, race/ethnicity, education, birthplace, smoking, and history of previous live birth; **^c^** adjusted for only population size of water district; **^d^** adjusted for only household income and folic acid intake; **^e^** “high mean atrazine” defined by as ≥95th percentile (0.14 µg/L) of mean values across all atrazine measurements in controls, 1999–2005; otherwise, “low mean atrazine”.

## Supplementary Materials

The following are available online at www.mdpi.com/1660-4601/14/8/889/s1, Table S1: Descriptive characteristics of cases and controls with available atrazine data, NBDPS, 1999–2005, Table S2: Descriptive characteristics of all potential controls with and without atrazine data in NBDPS, 1999–2005.
