# Supplementary material for: Estimated Maternal Pesticide Exposure from Drinking Water and Heart Defects in Offspring
Source: Int J Environ Res Public Health. 2017 Aug 8;14(8):889. doi: 10.3390/ijerph14080889 (PMC5580593; doi:10.3390/ijerph14080889)
Supplement: Supplementary file 1 [file ijerph-14-00889-s001.pdf]

Supplementary Materials: Estimated Maternal Pesticide Exposure from Drinking Water and Heart Defects in Offspring

Jihye Kim, Michael D. Swartz, Peter H. Langlois, Paul A. Romitti, Peter Weyer, Laura E. Mitchell, Thomas J. Luben, Anushuya Ramakrishnan, Sadia Malik, Philip J. Lupo, Marcia L. Feldkamp, Robert E. Meyer, Jennifer J. Winston, Jennita Reefhuis, Sarah J. Blossom, Erin Bell, and A. J. Agopian * and the National Birth Defects Prevention Study

Table S1. Descriptive characteristics of cases and controls with available atrazine data, NBDPS [a], 1999–2005.

	Cases		Controls		*p*-Value [c]
	N	%	N	%	
Characteristics [b]	1620	100.0	1335	100.0	
State					< 0.001
Arkansas	540	33.3	349	26.1	
Iowa	475	29.3	442	33.1	
North Carolina	196	12.1	228	17.1	
Utah	409	25.3	316	23.7	
Delivery year					0.001
1999 [d]	138	8.5	66	4.9	
2000 [d]	126	7.8	137	10.3	
2001 [d]	122	7.5	107	8.0	
2002 [d]	147	9.1	104	7.8	
2003	365	22.5	330	24.7	
2004	382	23.6	328	24.6	
2005	340	21.0	263	19.7	
Infant sex					0.213
Male	851	52.6	671	50.3	
Female	768	47.4	664	49.7	
Plurality of pregnancy					<0.001
Yes	102	6.3	34	2.6	
No	1517	93.7	1300	97.5	

Previous live birth					0.611
Yes	968	59.8	810	60.7	
No	652	40.3	525	39.3	
Maternal age					0.006
<20	142	8.8	123	9.2	
20–24	438	27.0	382	28.6	
25–29	497	30.7	400	30.0	
30–34	352	21.7	287	21.5	
35–39	148	9.1	133	10.0	
≥40	43	2.7	10	0.8	
Maternal race/ethnicity					0.783
Non-Hispanic White	1232	76.2	1012	76.2	
Non-Hispanic Black	174	10.8	135	10.2	
Hispanic	118	7.3	109	8.2	
Other	92	5.7	73	5.5	
Mother's education					0.429
Less than high school	218	13.5	174	13.1	
High school	454	28.1	349	26.2	
Greater than high school	946	58.5	810	60.8	
Household income					0.708
<$10,000	244	15.7	187	14.6	
$10,000–$50,000	418	26.9	354	27.7	
>$50,000	894	57.5	736	57.6	
Mother's birthplace in U.S.					0.069
Yes	1461	90.3	1176	88.2	
No	157	9.7	157	11.8	
Cigarette smoking during pregnancy					0.749
Yes	378	23.4	305	22.9	
No	1240	76.6	1029	77.1	
Maternal gestational or pregestational diabetes					0.004
Yes	176	10.9	103	7.7	

No	1441	89.1	1,230	92.3	
Water district population size					0.001
< 5000	235	14.5	208	15.6	
5000–200,000	1051	65.0	922	69.1	
> 200,000	332	20.5	205	15.4	
Periconceptional Folic acid intake[e]					0.609
Yes	895	55.3	725	54.3	
No	725	44.8	610	45.7	

NBDPS, National Birth Defects Prevention Study; [a] Included NBDPS data from Arkansas, Iowa, North Carolina, and Utah; [b] Some characteristic counts do not sum to the totals due to missing data; [c] Estimated using chi-square test; [d] Data from North Carolina and Iowa were not available for deliveries in 1999–2002; [e] Between 1 month before pregnancy and 1st month of pregnancy.

Table S2. Descriptive characteristics of all potential controls with and without atrazine data in NBDPS [a], 1999–2005.

	**Atrazine Data**		**Missing Atrazine Data**		**OR (95% CI)**	*p*-Value [c]
	**N**	**%**	**N**	**%**		
Characteristic [b]	1335	100.0	322	100.0		
State						<0.001
Arkansas	349	26.1	174	54.0	15.76 (8.18–30.34)	
Iowa	442	33.1	82	25.5	5.86 (2.99–11.48)	
North Carolina	228	17.1	56	17.4	7.76 (3.88–15.54)	
Utah	316	23.7	10	3.1	Ref.	
Delivery year						<0.001
1999 [d]	66	4.9	17	5.3	Ref.	
2000 [d]	137	10.3	53	16.5	1.50 (0.81–2.79)	
2001 [d]	107	8.0	34	10.6	1.23 (0.64–2.38)	
2002 [d]	104	7.8	34	10.6	1.27 (0.66–2.45)	
2003	330	24.7	53	16.5	0.62 (0.34–1.14)	
2004	328	24.6	54	16.8	0.64 (0.35–1.17)	
2005	263	19.7	77	23.9	1.14 (0.63–2.05)	
Infant sex						0.829
Male	671	50.3	164	50.9	Ref.	

Female	664	49.7	158	49.1	0.97 (0.76–1.24)	
Plurality of pregnancy						0.577
Yes	34	2.6	10	3.1	1.23 (0.60–2.51)	
No	1300	97.5	312	96.9	Ref.	
Previous live birth						0.655
Yes	810	60.7	191	59.3	0.95 (0.74–1.21)	
No	525	39.3	131	40.7	Ref.	
Maternal age						0.001
<20	123	9.2	35	10.9	1.13 (0.73–1.74)	
20–24	382	28.6	62	19.3	0.64 (0.46–0.91)	
25–29	400	30.0	101	31.4	Ref.	
30–34	287	21.5	87	27.0	1.20 (0.87–1.66)	
35–39	133	10.0	29	9.0	0.86 (0.55–1.36)	
≥40	10	0.8	8	2.5	3.17 (1.22–8.23)	
Maternal race/ethnicity						0.682
Non-Hispanic White	1012	76.2	238	74.4	Ref.	
Non-Hispanic Black	135	10.2	40	12.5	1.26 (0.86–1.84)	
Hispanic	109	8.2	25	7.8	0.98 (0.62–1.54)	
Other	73	5.5	17	5.3	0.99 (0.57–1.71)	
Mother's education						0.606
Less than high school	174	13.1	47	14.6	1.22 (0.82–1.84)	
High school	349	26.2	77	23.9	Ref.	
Greater than high school	810	60.8	198	61.5	1.11 (0.83–1.48)	
Household income						0.001
<$10,000	187	14.6	45	15.1	1.27 (0.88–1.85)	
$10,000–$50,000	354	27.7	115	38.5	1.72 (0.30–2.27)	
>$50,000	736	57.6	139	46.5	Ref.	
Mother's birthplace in U.S.						0.764
Yes	1176	88.2	286	88.8	Ref.	
No	157	11.8	36	11.2	0.94 (0.64–1.39)	
Cigarette smoking during pregnancy						0.162

Yes	305	22.9	62	19.3	0.81 (0.59–1.09)	
No	1029	77.1	260	80.8	Ref.	
Maternal gestational or pregestational diabetes						0.006
Yes	103	7.7	11	3.4	0.42 (0.22–0.80)	
No	1230	92.3	311	96.6	Ref.	
Water district population size						<0.001
< 5000	208	15.6	85	28.4	Ref.	
5000–200,000	922	69.1	214	71.6	0.57 (0.42–0.76)	
>200,000	205	15.4	0	0.0	NA	
Periconceptional Folic acid intake [e]						0.187
Yes	725	54.3	188	58.4	1.18 (0.92–1.51)	
No	610	45.7	134	41.6	Ref.	

NBDPS, National Birth Defects Prevention Study; OR, (crude) odds ratio for missing atrazine; CI, confidence interval; NA, not applicable; [a] Included NBDPS data from Arkansas, Iowa, North Carolina, and Utah; [b] Some characteristic counts do not sum to the totals due to missing data; [c] Estimated using chi-square test; [d] Data from North Carolina and Iowa were not available for deliveries in 1999–2002; [e] Between 1 month before pregnancy and 1st month of pregnancy.

© 2017 by the authors; licensee MDPI, Basel, Switzerland. This article is an open access article distributed under the terms and conditions of the Creative Commons by Attribution (CC-BY) license (http://creativecommons.org/licenses/by/4.0/).
